# Effects of Nutritional Interventions in Older Adults with Malnutrition or at Risk of Malnutrition on Muscle Strength and Mortality: Results of Pooled Analyses of Individual Participant Data from Nine RCTs

**DOI:** 10.3390/nu15092025

**Published:** 2023-04-22

**Authors:** Judith I. van Zwienen-Pot, Ilse Reinders, Lisette C. P. G. M. de Groot, Anne Marie Beck, Ilana Feldblum, Inken Jobse, Floor Neelemaat, Marian A. E. de van der Schueren, Danit R. Shahar, Ellen T. H. C. Smeets, Michael Tieland, Hanneke A. H. Wijnhoven, Dorothee Volkert, Marjolein Visser

**Affiliations:** 1 Research Centre Innovations in Care, Rotterdam University of Applied Sciences, 3015 EK Rotterdam, The Netherlands; 2 Department of Nutrition and Dietetics, Amsterdam University Medical Centers, Location VUmc, 1081 HV Amsterdam, The Netherlands; 3 Department of Health Sciences, Faculty of Science, Amsterdam Public Health Research Institute, Vrije Universiteit, 1081 HV Amsterdam, The Netherlandshanneke.wijnhoven@vu.nl (H.A.H.W.); m.visser@vu.nl (M.V.); 4 Division of Human Nutrition and Health, Wageningen University & Research, 6703 HE Wageningen, The Netherlands; lisette.degroot@wur.nl (L.C.P.G.M.d.G.); marian.devanderschueren@han.nl (M.A.E.d.v.d.S.);; 5 The Dietitians and Nutritional Research Unit, EATEN, Herlev and Gentofte Hospital, DK-2730 Herlev, Denmark; anne.marie.beck@regionh.dk; 6 The Daniel Abraham International Center for Health Nutrition, Department of Public Health, Faculty of Health Sciences, Ben-Gurion University of the Negev, Beersheba 84105, Israeldshahar@bgu.ac.il (D.R.S.); 7 Institute for Biomedicine of Aging, Friedrich-Alexander-Universität Erlangen-Nürnberg, 90408 Nuremberg, Germanydorothee.volkert@fau.de (D.V.); 8 Department of Nutrition, Dietetics and Lifestyle, HAN University of Applied Sciences, 6525 EN Nijmegen, The Netherlands; 9 Center of Expertise Urban Vitality, Amsterdam University of Applied Science, 1067 SM Amsterdam, The Netherlands; m.tieland@hva.nl

**Keywords:** dietary counseling, nutritional support, nutritional therapy, undernutrition, older adults

## Abstract

Nutritional intervention studies in older adults with malnutrition aim to improve nutritional status. Although these studies show a significant gain in body weight, there is inconsistent evidence of clinical effectiveness on muscle strength and mortality. This study aimed to examine the effects of nutritional interventions on muscle strength and risk of mortality in older adults (malnourished or at risk) and explore whether these effects are influenced by participant characteristics. Individual participant data were used from nine RCTs (community setting, hospital and long-term care; duration 12–24 weeks and included oral nutritional supplements, dietary counseling, or both). Handgrip strength (HGS) was measured in seven RCTs and six RCTs obtained mortality data. A ≥3 kg increase in HGS was considered clinically relevant. Logistic generalized estimating equations analyses (GEE) were used to test intervention effectiveness. GEE showed no overall treatment effect (OR 1.11, 95% CI 0.78–1.59) on HGS. A greater, but not statistically significant, effect on HGS was observed for older (>80 years) versus younger participants. No significant treatment effect was observed for mortality (OR 0.78, 95% CI 0.42–1.46). The treatment effect on mortality was greater but remained non-significant for women and those with higher baseline energy or protein intake. In conclusion, no effects of nutritional interventions were observed on HGS and mortality in older adults (malnourished or at risk). While the treatment effect was modified by some baseline participant characteristics, the treatment also lacked an effect in most subgroups.

## 1. Introduction

While malnutrition, a serious problem associated with poor health [1], is present in all age groups, prevalence rates are particularly high among older adults [2,3,4,5]. Malnutrition is associated with several adverse clinical outcomes such as poor quality of life, increased morbidity and mortality, higher healthcare costs, lower muscle strength, and muscle mass, and reduced functional status [1,6,7,8,9]. These negative outcomes emphasize the importance of recognizing and treating older adults with malnutrition and those at risk timely and effectively. Most preventive or treatment intervention studies in older adults with malnutrition and those at risk, provide nutritional support (extra energy and protein) through subscribing to oral nutritional supplements (ONS), dietary counseling, or a combination of both. So far, intervention studies have shown positive treatment effects on outcome measures such as body weight and energy and/or protein intake [2,4,10]. However, there is inconsistent evidence of clinical effectiveness on other patient-relevant outcomes, including muscle strength and mortality [2,4,11,12,13,14,15]. This heterogeneity can be explored by applying individual participant data (IPD) meta-analysis [16].

As part of the Joint Program Initiative Malnutrition in the Elderly (MaNuEL) Knowledge Hub [17], we pooled individual participant data from nine nutritional intervention RCTs conducted in older adults with malnutrition or at risk of malnutrition. Individual participant data (IPD) allows the analyses to be standardized across trials. It makes the direct derivation of information possible, regardless of how it was reported in the original trials [16,18,19]. The results for specific subgroups of participants can be obtained across trials and differential treatment effects can be obtained across individual participant characteristics [16], which can provide insight into which type of intervention is effective in whom.

Our previous study showed the effectiveness of the interventions on body weight and energy intake using IPD [10]. We wanted to explore whether this effect would extrapolate to other patient-relevant measures available in the same data from older adults with malnutrition or at risk. Hence, handgrip strength (as a proxy for muscle strength [20,21,22]) and mortality were selected. Both outcome measures are generally measured in research [23,24,25,26] and, thus we expected it to be widely available in these datasets. Therefore, we explored the effect of the nutritional intervention on muscle strength and risk of mortality using IDP analysis and whether the effects were influenced by patient characteristics.

## 2. Materials and Methods

### 2.1. Study Selection

The study selection for this study is according to the same approach as the study selection of the earlier presented MaNuEL study published in 2019 [10]. To identify trials that could be used to investigate the effect on body weight and energy intake [10], the search strategy for nutritional intervention trials among older adults was based on two published reviews by Milne et al. [2] and de van der Schueren et al. [11] In addition, the MEDLINE database was used with the search criteria “nutritional support”, “diet”, “malnutrition”, “undernutrition”, “protein-energy malnutrition” (Medical Subject Headings terms without language restrictions). Reference lists of found trials and reviews were searched for possible eligible RCTs. 

The inclusion criteria were as follows: randomized controlled trials, mean participant aged 55 years or older, body weight measured at baseline and follow-up, the intervention should have aimed to increase energy and/or protein intake through ONS, dietary counseling, or their combination. RCTs were excluded in the case of (partial) enteral or parenteral feeding, patients being treated for cancer, and when the nutritional intervention was combined with physical activity (and it was not possible to separate these intervention effects). A total of 38 nutritional intervention studies met the inclusion criteria. For eight studies, no contact information was available. Of the remaining 30 studies, the principal investigators were contacted for contribution (main reasons for not contributing were: no response (*n* = 6), data had been destroyed (*n* = 6), and not being able to provide the data (*n* = 5)). A flowchart of the trial selection can be found elsewhere [10]. Eventually, data from nine nutritional intervention studies with a total of 1265 participants were included, and subsequently, we checked which studies were suitable for pooling for data on handgrip strength (HGS) and mortality. In all studies, the procedures followed were in accordance with the ethical standards of their local ethical committee and all participants gave consent.

### 2.2. Study Setting and Type of Intervention

The included RCTs were categorized according to setting: (1) hospital, (2) community and (3) long-term care (i.e., homes for older adults, nursing homes, and mixed homes). The type of intervention was divided into (1) ONS only (2) dietary counseling only (3) a combination of ONS and dietary counseling.

### 2.3. Participant Data Extraction

Individual participant data were extracted from the nine included nutritional intervention RCTs. Data on baseline age (y), sex, measured body height (m), measured body weight (kg, baseline, and follow-up), maximum handgrip strength (kg, baseline, and follow-up), mortality, energy, and protein intake (kcal/day and g/day, baseline and follow-up) were used. Baseline body mass index (BMI) was calculated as body weight at baseline (kg) divided by body height (m^2^). To calculate grams of protein intake per kg body weight, body weight was adjusted to a BMI of 22–27 kg/m^2^ as described by Berner et al. [27]. Changes in body weight (kg), protein intake (grams per kg body weight per day), and energy intake (kcal per day) were calculated by subtracting the baseline value from the follow-up value. 

### 2.4. Muscle Strength

Handgrip strength (in kg) as a proxy for muscle strength [22] was applied to determine changes in maximum peripheral muscle function. Change in handgrip strength (HGS) during intervention was measured in eight RCTs. Seven RCTs measured HGS in kg [28,29,30,31,32,33,34] and one RCT in kPA [35]. Since the conversion of measurements in kPA to kg is not possible, data from the RCT of Stange et al. [35]. were eventually excluded from the HGS analyses. The maximum strength of two or three consecutive measurements of the dominant hand was used from six RCTs. From Schilp et al. [33] right-hand maximum strength was used, and from Neelemaat et al. [32] the maximum strength of the non-dominant hand was used. In most RCTs, HGS was measured with a Jamar Hydraulic Hand Dynamometer, except the RCT by Neelemaat et al. [32], which used a Baseline Hydraulic Hand Dynamometer. A handgrip strength of <20 kg for women and <30 kg for men was categorized as low [36,37]. 

To our knowledge no definition of a clinically relevant increase in HGS in older adults with malnutrition, or those at risk, is available. In the present study, we considered an increase of ≥3 kg between baseline and follow-up (follow-up duration ranged from 12 to 24 weeks) as clinically relevant. For the HGS dataset only participants with complete data on age, BMI at baseline and follow-up, and HGS at baseline and follow-up were included.

### 2.5. Mortality

Mortality data were obtained from six RCTs [28,29,32,33,35,38]. Since the exact follow-up time, until death was not available from all RCTs, mortality was used as a dichotomous variable. Data on mortality either during the intervention period (intervention duration ranged from 12 to 24 weeks) [32,33,35,38] or up to 6 months after the start of the intervention (intervention duration was 12 weeks) were used [28,29]. For the mortality dataset, only participants with complete data on age, BMI at baseline, and mortality status either during intervention or up to six months after the start of the intervention were included.

### 2.6. Statistical Analysis

Descriptive statistical methods were used to express means, standard deviations, percentages, and frequencies to describe participants’ characteristics at baseline. Differences at baseline between intervention and control were tested using a *t*-test for r continuous variables and Chi-square tests for categorical variables. Logistic generalized estimating equations analyses (GEE) with an exchangeable correlation structure were used to test the effect of the nutritional intervention on handgrip strength and mortality. This analysis technique is suitable for the longitudinal analysis of dichotomous outcome variables while accounting for the clustering of participants within the RCTs. 

For both outcome variables (HGS and mortality), a crude model was built (i.e., difference between intervention and control group on average while controlling for clustered data). Adjusted models were also built (i.e., the crude model including adjustment for potential confounders). In the models for HGS, adjustments were made for age, sex, BMI at baseline, and HGS at baseline. In the models for mortality, adjustments for age, sex, and BMI at baseline were made. 

To investigate whether the intervention effects differed according to patient characteristics, predefined subgroup analyses were performed for the primary outcome measures according to age, sex, baseline handgrip strength, baseline BMI, baseline energy intake, and baseline protein intake per kg body weight. An interaction term for the treatment group versus each of these characteristics was included in the model. When the interaction term was significant (*p* < 0.10), the sample was stratified according to the relevant baseline characteristic (age younger than 80 years or 80 years and older, normal and low HGS (low: <20 kg for women and <30 kg for men), normal and low BMI (low: <22 kg/m^2^) [39], normal and low protein intake (low: <0.8 g per kg body weight per day). Energy intake was stratified at the baseline median of the sample (1682 kcal/day for the HGS dataset and 1460 kcal/the day for mortality dataset). The same models were again run for each stratum and adjusted for baseline variables. 

To investigate whether the intervention effect differed according to the changes in body weight, energy intake, and protein intake due to the intervention, predefined subgroup analyses were performed for the outcome measure increase of ≥3 kg in HGS according to the previously used variables meaningful weight gain (≤1.0 kg versus >1.0 kg), and meaningful change in energy intake (≤250 kcal/day versus >250 kcal/day) [10] and increase in protein intake, based on median change, <4 g versus ≥4 g/day during the intervention. These analyses could not be performed for the outcome mortality due to the smaller number of events (deceased). 

An increase of ≥3 kg in HGS between baseline and follow-up was considered clinically relevant, but because of this rather arbitrary definition, sensitivity analyses were performed using ≥2 kg and ≥4 kg to indicate an increase in HGS. 

Intervention effects on HGS and mortality are presented as odds ratios (OR) with 95% confidence intervals (CIs). A *p* value of <0.05 was considered statistically significant for descriptive statistics and GEE analyses. All analyses were performed using IBM SPSS statistics 22.0 (Armonk, NY, USA: IBM Corp.).

## 3. Results

### 3.1. Study Setting and Type of Intervention

Four RCTs were performed in a hospital setting including older adults at discharge from the hospital [28,29,32,38], three were performed among community-dwelling older adults [30,33,34], and two in long-term care [31,35], (Supplemental Table S1). Of the three categories of intervention strategies included, four RCTs provided ONS only [30,31,34,35] and four RCTs used dietary counseling (ONS were provided only if regular intake was inadequate) [28,29,33,38]. The combination of ONS and dietary counseling was used in one RCT [32]. 

### 3.2. Participants

The nine selected nutritional intervention RCTs provided data from 1265 participants for possible inclusion. The dataset used for the HGS analyses included 7 RCTs [28,29,30,31,32,33,34] and the second dataset used for the mortality analyses included 6 RCTs [28,29,32,33,35,38].

For the HGS dataset only participants with complete data on age, BMI at baseline and follow-up, and HGS at baseline and follow-up were included. For this reason, 250 participants had to be excluded and this resulted in a pooled dataset of 669 participants with mean age of 79.6 ± 8.2 years and 63.1% women (Table 1). At baseline, the mean BMI was 23.2 ± 4.1 kg/m^2^ and a total of 270 participants (40.4%) had a BMI (<22 kg/m^2^). The majority of the participants (65.5%) had low handgrip strength. Participants in the intervention group were more likely to be institutionalized compared to those in the control group (*p*-value = 0.001) because of an unequal distribution of participants in the intervention group in one study conducted in long-term care [31]. There were no further statistically significant differences between participants in the intervention group compared to those in the control group at baseline in the HGS dataset. 

For the mortality dataset, only participants with complete data on age, BMI at baseline, and mortality status either during intervention or up to six months after the start of the intervention were included. Therefore,163 participants with lacking data were excluded. Eventually, the pooled dataset consisted of 762 participants with a mean baseline age of 78.8 ± 8.5 years and 64.4% women (Table 1) The Mean BMI at baseline was 23.2 ± 4.9 kg/m^2^ and 45.3% had a BMI (<22 kg/m^2^). Participants in the intervention group had a higher baseline energy intake compared to the control group (*p* = 0.049). There were no further significant differences between participants in the intervention group compared to those in the control group at baseline in the mortality dataset.

### 3.3. Treatment Effect on Handgrip Strength

**Figure 1:**
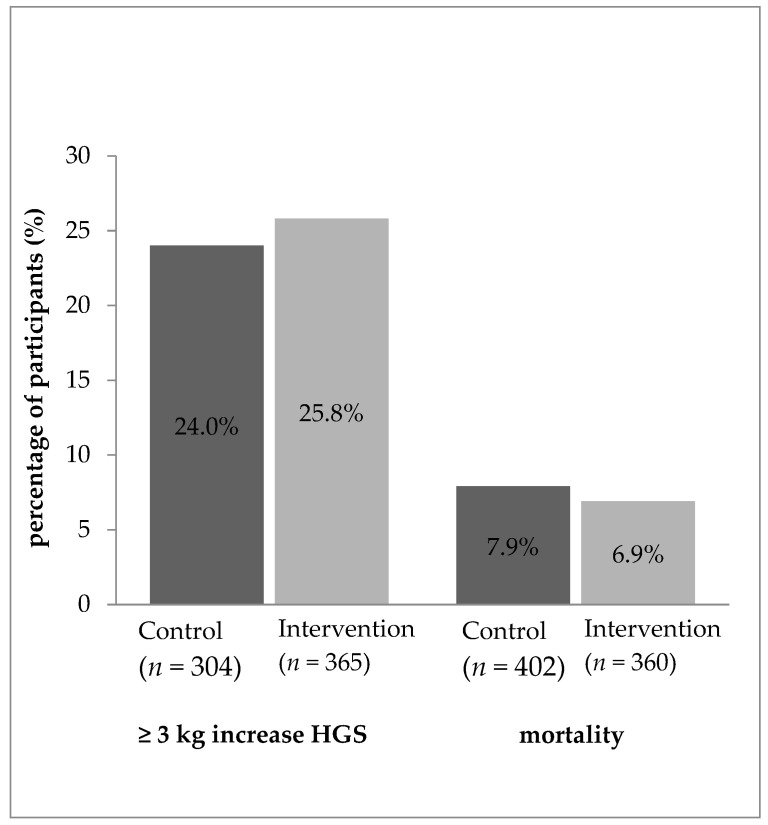
Effect of nutritonal intervention on ≥3 kg increase in HGS and mortaliy for older adults with (risk of) malnutrition: results from 9 pooled RCTs.

Of all 669 participants in the HGS dataset, 167 (25%) had a clinically relevant increase of ≥3 kg between baseline and follow-up in HGS. In the intervention group, 94 participants (25.8%) experienced a clinically relevant increase in HGS versus 73 participants (24%) in the control group (Figure 1). 

The mean change in HGS in kg was 0.24 (±4.1) for the intervention group and −0.06 (±4.4) for the control group (*p* = 0.370). The number of participants with weight gain (>1.0 kg) was higher in the intervention group (*n* = 158, 43.3%) compared to the control group (*n* = 103, 33.9%, *p* = 0.014). Mean energy intake increased by 199 ± 613 kcal/day in the intervention group and by 46 ± 540 kcal/day in the control group (*p* = 0.003). Mean protein intake increased by 8.8 ± 26.7 g/day in the intervention group and by 1.3 ± 25 g/day in the control group (*p* = 0.001) (Table 2). 

The overall treatment effects derived from GEE analyses, both crude and adjusted for several baseline variables, are shown in Table 3. The treatment effect (adjusted for several baseline variables) on a clinically relevant increase of ≥3 kg HGS between baseline and follow-up was OR 1.11 with a 95% CI of 0.78–1.59.

Predefined subgroup analyses derived from GEE analyses are shown in Table 4. The treatment effect on HGS was modified by age (*p* = 0.10) and tended to be modified by protein intake at baseline (*p* = 0.12) but was not modified by sex, BMI, handgrip strength, or energy intake at baseline. The treatment effect in participants aged ≥80 years was OR 1.35 (0.93–1.95) and in those aged <80 years OR 0.94 (0.63–1.40). In participants with a higher baseline protein intake (≥0.8 g per kg body weight per day,) the treatment effect on ≥3 kg increase in HGS was OR 1.91 (95% CI 1.23–2.95) and statistically significant while no positive treatment effect for those with a low protein intake (<0.8 g per kg body weight) was observed (OR 0.64 (0.37–1.10)), suggesting that the treatment effect on HGS was greater in those who met the protein requirement at baseline. 

The treatment effect (adjusted for several baseline variables) on a clinically relevant increase in HGS of participants who had gained more than 1 kg of body weight at follow-up (282 participants, 42.2%) was OR 0.79 (0.39–1.63) and was OR 1.32 (0.92–1.90) for those who gained 1 kg or less. The treatment effect was OR 1.17 (0.70–1.97) in participants with an increase in energy intake of ≥250 kcal/day at follow-up and OR 1.33 (0.83–2.13) for those with a change in energy intake of <250 kcal/day. Furthermore, in participants with an increase in protein intake (≥4 g/day) at follow up the treatment effect was OR 1.16 (0.62–2.16) and OR 1.33 (0.65–2.72) for those with a smaller increase in protein intake at follow-up. 

In sensitivity analyses, in which an increase of ≥2 kg, respectively, ≥4 kg in HGS between baseline and follow-up was considered clinically relevant, the results were comparable to using a ≥3 kg increase as clinically relevant. The adjusted overall treatment effect for an increase of ≥2 kg in HGS was OR 1.150 with a 95% CI of 0.81–1.63 and for ≥4 kg OR 1.11 (0.79–1.58). 

### 3.4. Treatment Effect on Mortality

Of the 762 participants in the mortality dataset, 57 (7.5%) were deceased during follow-up. In the control group, 32 (8%) participants were deceased versus 25 (6.9%) participants in the intervention group (Figure 1). The follow-up characteristics of the participants stratified by treatment group are shown in Table 2. The number of participants with weight gain (>1.0 kg) was higher in the intervention group (*n* = 163, 45.3%) compared to the control group (*n* = 137, 34.1%, *p* ≤ 0.001). Mean energy intake increased by 239 ± 664 kcal/day in the intervention group and by 105 ± 542 kcal/day in the control group (*p* = 0.010). Mean protein intake increased by 9.1 ± 29.6 g/day in the intervention group and by 3.5 ± 26.7 g/day in the control group (*p* = 0.001). 

The overall treatment effect on mortality, derived from the fully adjusted GEE analyses was OR 0.78 (95% CI 0.42–1.46), suggesting a protective effect of the nutritional intervention on mortality, although not statistically significant (Table 3). The treatment effect on mortality was not modified by age, BMI, or handgrip strength at baseline, but was modified by sex (*p* = 0.07), baseline energy intake (*p* = 0.004) and baseline protein intake (*p* = 0.052). The treatment effect for women (OR 0.63 (0.34–1.17) was greater compared to the effect in men (OR 1.13 (95% CI 0.60–2.10)). Furthermore, the treatment effect was greater in participants with a higher baseline energy intake (≥1460 kcal/day OR 0.76 (95% CI 0.34–1.68) compared to those with a baseline energy intake below the median (OR 1.09 (95% CI 0.45–2.66)). Finally, the treatment effect was greater in participants with a higher baseline protein intake (≥0.8 g protein per kg body weight per day, OR 0.54 (95% CI 0.21–1.36)) as compared to those with a lower baseline protein intake (OR 1.73 (95% CI 0.64–4.64)). However, consistent with the results in the complete sample, in all subgroups, the treatment effect on mortality remained not statistically significant. 

## 4. Discussion

To our knowledge, this is the first study using pooled individual participant data of multiple nutritional intervention RCTs conducted in older adults with malnutrition, or at risk, examining the overall effect of the nutritional intervention on handgrip strength and risk of mortality. Its results showed no beneficial effect of nutritional intervention by oral nutritional supplements and/or dietary counseling in older adults with malnutrition and those at risk, on change in handgrip strength and mortality risk. 

Some previous systematic reviews and meta-analyses also reported no positive effects of nutritional intervention on handgrip strength in older adults [2,12,13]. These results, combined with our findings, therefore suggest that older persons with malnutrition and those at risk may not benefit from a nutritional intervention with regard to handgrip strength. On the other hand, there are also studies reporting positive effects, such as the systematic review that examined studies conducted in nursing homes [40]. We cannot exclude that handgrip strength is less sensitive to intervention changes than other measures of muscle strength. For example, exercise intervention studies conducted in older adults have shown improvements in leg extension strength but not in handgrip strength [41]. Future nutritional intervention studies should include additional measures of muscle strength to evaluate treatment effects on different measures of muscle strength in older adults. Yet, it remains important to include HGS at study baseline in future intervention studies. Measurement of handgrip strength is widely applicable and non-invasive and handgrip strength is an important prognostic factor for relevant clinical outcomes [42]. 

Our results on mortality confirm the results of a previous meta-analysis showing no reduction in mortality risk in the intervention group compared with controls [2]. In that meta-analysis, a reduction in mortality was observed when limiting the trials to those conducted in participants defined as undernourished or at risk of undernutrition at baseline (RR 0.79, 95% CI 0.64 to 0.97). However, when performing subgroup analyses in our study in participants with a low BMI (<22 kg/m^2^) or a low protein and energy intake at baseline, still no significant effect on mortality was observed (OR ranged from 0.59–1.73). In contrast, the Kaegi-Braun 2021 meta-analysis found a decrease in mortality among patients receiving nutritional support compared to those who did not receive it, with an odds ratio of 0.72 (95% CI 0.57 to 0.91, *p* = 0.006). This meta-analysis was conducted among hospitalized medical patients, which could account for some of the discrepancies between our findings and their results, given the wider scope of our study sample. Furthermore, the higher age of our study sample compared to the age group in their study could also contribute to the difference in our results.

The strengths of this study lie in its use of a large pooled dataset of individual participant data from nine RCTs covering a variety of settings, treatments, and participants, as well as the use of two clinically relevant outcomes. A unique aspect of this study was the ability to examine potential baseline patient characteristics that could modify the treatment effect. Treatment effect on HGS was modified by age and tended to be modified by protein intake per kg body weight at baseline; a higher age and a higher protein intake at baseline resulted in a greater effect on increasing handgrip strength. Furthermore, the treatment effect on mortality was greater for women and those with higher energy or protein intake per kg body weight at baseline. However, the treatment effects within these subgroups remained not statistically significant, with one exception: in participants with a higher protein intake (≥0.8 g protein per kg body weight per day) at baseline, the treatment effect on ≥3 kg increase in HGS was statistically significant. It is striking that older adults who eat more protein than the current RDA and relatively more energy at baseline show a greater treatment effect for both HGS and mortality, while the results of the analyses incorporating the actual measured change in body weight or change in energy or protein intake during the intervention did not impact the effect on increasing HGS. The reasons for these results are unclear but may suggest that the impact of nutritional interventions is influenced by baseline nutritional status. Indeed, Verlaan et al. [43] showed that sufficient protein intake at baseline positively influenced an increase in muscle mass in older adults who received the nutritional intervention. 

A possible explanation for this observation could be that participants with a higher energy and protein intake at baseline were in a relatively better health condition and therefore had a better chance of recovery, thereby positively affecting HGS and mortality risk. Furthermore, it can be hypothesized that participants with a higher energy and protein intake at baseline might have been better able to increase their energy intake (and thus gain weight) as a result of the intervention. However, the treatment effect on gaining >1 kg body weight or increasing >250 kcal in energy intake during the intervention was not different between participants with a lower or higher energy intake at baseline [10]. 

It is possible that some aspects of our search strategy and the interventions used in the RCTs we included may have played a role in the absence of a treatment effect in our study. Of the 38 identified articles, only data from nine could be obtained, as the data from the remaining articles was inaccessible due to either lack of response or because the data had already been destroyed, which could lead to a potential selection bias. 

An additional limitation of our study is the lack of a definition for a clinically relevant increase in HGS in older adults with malnutrition or at risk. However, in the sensitivity analyses, different definitions were used which all resulted in the same conclusion. 

The variations in interventions can also be viewed as a limitation. As the interventions all had some differences and we cannot exclude the possibility that a particular type of intervention may perform better than the others. The interventions in the included RCTs all aimed to increase energy and/or protein intake through dietary counseling, ONS, or their combination. However, some studies also had additional aims, such as an increase in micronutrient intake [30] which can contribute to variation on effects. In addition, variations in the method used to assess malnutrition may have influenced the intervention effects. Future studies should aim to include homogeneous criteria, for example the GLIM criteria.

Compliance was available in the studies providing ONS alone and in one study in which ONS in combination with dietary counseling was used (Supplemental Table S1). Therefore, stratification by high compliance was not possible in the current study. It is valuable information to know whether the included studies were able to reach their intervention goals. Even though energy intake may increase due to the intervention, the total intake may still remain below energy requirements, making it unlikely that there will be a positive effect on outcomes [11,44,45]. Underfeeding is indeed not likely to elicit clinically beneficial outcomes.

Stratification on setting (hospital, community-dwelling, and institutionalized) and treatment characteristics, such as duration of treatment and intervention type, on clinical outcomes, was not possible in the current study, because, for example in the HGS dataset, there was only one study conducted among institutionalized participants and only one study using ONS combined with dietary counseling. Future pooled analyses should address the influence of study characteristics, compliance, and reaching intervention goals, on clinical outcomes.

The heterogeneity of outcomes complicates the pooling of individual patient data and shows the necessity for harmonized research and the development of a minimum dataset for nutritional intervention studies in older adults with malnutrition and those at risk [23]. More high-quality nutritional intervention studies in older persons at risk of malnutrition are needed using different types of treatment strategies in different settings using similar clinically relevant outcomes to enable statistical analyses using individual patient data in the future. MaNuEL researchers are presently working on a Core Outcome Set to establish agreement on a set of relevant clinical outcomes for different contexts to be used in future nutritional intervention studies treating malnutrition in older persons [23].

## 5. Conclusions

In summary, our results based on a pooled dataset of individual participant data from 9 RCTs did not show beneficial effects of a nutritional intervention (dietary counseling, oral nutritional supplements, of the combination of both) on handgrip strength and mortality in older persons with malnutrition or at risk. While the treatment effect was modified by some baseline patient characteristics, in general, the treatment lacked an effect in most subgroups. Future harmonized research is required to determine who will benefit from what specific intervention and in which setting to effectively improve health and functioning in older individuals with malnutrition and those at risk.

## Figures and Tables

**Table 1:** Baseline characteristics of older adults with (risk of) malnutrition, who participated in the RCTs: pooled participant data from 9 nutritional intervention RCTs, stratified by treatment group.

Baseline Characteristics	Handgrip Strength			Mortality		
	All (*n* = 669)	Control (*n* = 304)	Intervention (*n* = 365)	All (*n* = 762)	Control (*n*= 402)	Intervention (*n* = 360)
Age (y) mean (±SD)	79.6 (±8.2)	79.6 (8.4)	79.5 (8.1)	78.8 (8.5)	78.4 (8.5)	79.3 (8.6)
Sex *n* (% women)	422 (63.1)	188 (61.8)	234 (64.1)	491 (64.4)	256 (64.4)	232 (64.4)
HGS (kg) mean (±SD)	20.5 (±8.6)	20.9 (8.3)	20.1 (8.8)	19.6 (8.4) ^a^	19.8 (±8.0)	19.5 (±8.7)
Normal HGS *n* (%) ^b^	231 (34.5)	109 (35.9)	122 (33.4)	140 (29.9)	66 (28.7)	74 (31.1)
Low HGS *n* (%)	438 (65.5)	195 (64.1)	243 (66.6)	328 (70.1)	164 (71.3)	164 (68.9)
BMI (kg/m^2^) Mean (±SD)	23.2(±4.1)	23.0 (4.0)	23.4 (4.3)	23.2 (4.9)	23.4 (5.1)	22.9 (4.7)
BMI < 20 *n* (%)	270 (40.4)	127 (41.8)	143 (39.2)	345 (45.3)	177(44.0)	168 (46.7)
BMI > 22 *n* (%)	399 (59.6)	177(58.2)	222 (60.8)	417 (54.7)	225 (56.0)	192 (53.3)
Setting						
Hospital *n* (%)	291 (43.5)	139 (45.7)	152 (41.6)	546 (71.7)	298 (74.1)	248 (68.9)
Community dwelling *n* (%)	249 (37.2)	125 (41.1)	124 (34.0)	129 (16.9)	62 (15.4)	67 (18.6)
Institutionalized *n* (%)	129 (19.3) ^c^	40 (13.2)	89 (24.4)	87 (11.4)	42 (10.4)	45 (12.5)
Intervention type						
Dietary counselling *n* (%)	-	-	146 (40.0)	-	-	228 (63.3)
ONS *n* (%)	-	-	156 (42.7)	-	-	45 (12.5)
Dietary counselling + ONS *n* (%)	-	-	63 (17.3)	-	-	87 (24.2)
Energy intake (kcal/day) mean (±SD) ^d^	1707 (558)	1688 (525)	1727 (589)	1491 (574) ^c^	1449 (552)	1539 (594)
Protein intake (g/kg/bw) mean (±SD) ^d,e^	1.00(0.38)	1.00(0.38)	1.00(0.38)	0.91 (0.42)	0.90 (0.41)	0.94 (0.43)
protein < 0.8 g/kg/bw *n* (%) ^e^	158 (30.2)	75 (28.8)	83 (31.4)	258 (41.1)	142 (42.9)	116 (39.2)
protein ≥ 0.8 g/kg/bw *n* (%) ^e^	366 (69.8)	185 (71.2)	181 (68.6)	369 (48.4)	189 (57.1)	180 (60.8)

^a^ Data available for *n* = 230 control and *n* = 238 intervention. ^b^ Cut-off values low HGS < 20 kg for women and <30 kg for men. ^c^ Statistical significant difference between intervention and control. ^d^ HGS dataset: data available for *n* = 260 control and *n* = 264 intervention; Mortality dataset: data available for *n* = 331 control and *n* = 296 intervention. ^e^ bodyweight was corrected to a BMI 22–27 kg/m^2^. Abbreviations: SD = standard deviation HGS = handgrip strength, BMI = body mass index, ONS = oral nutritional support, g/kg/bw = gram per kilogram bodyweight.

**Table 2:** Follow-up and change values of older adults with (risk of) malnutrition, who participated in the RCTs: pooled participant data from 9 nutritional intervention RCTs, stratified by treatment group.

	Handgrip Strength			Mortality		
Follow-Up Values	All (*n* = 669)	Control (*n* = 304)	Intervention (*n* = 365)	All (*n* = 762)	Control (*n*= 402)	Intervention (*n* = 360)
HGS (kg) mean (±SD) ^a^	20.6 (8.9)	20.9 (8.6)	20.3 (9.2)	20.5 (8.6) ^a^	20.8 (8.2)	20.2 (8.9)
Absolute change in HGS (kg) mean (±SD)	0.1 (4.2)	−0.1 (4.4)	0.2 (4.1)	0.3 (4.6)	0.2 (4.9)	0.3 (4.3)
Energy intake (kcal/day) mean (±SD) ^b,c^	1840 (548) ^b^	1738 (511)	1941 (565)	1669 (614) ^b^	1554 (556.0)	1803 (652)
Protein intake (g/day) mean (±SD) ^b,c^	70.5 (23.7) ^b^	66.4 (22.1)	74.5 (24.6)	65.7 (24.9) ^b^	61.6 (23.0)	70.5 (26.2)
Absolute change in energy intake (kcal/day) mean (±SD) ^d^	123 (582) ^b^	46 (540)	199 (613)	168 (606) ^b^	105 (542)	239 (664)
Absolute change in protein intake (g/day) mean (±SD) ^d^	5.0 (26.3) ^b^	1.3 (25)	8.8 (26.7)	6.2 (28.1) ^b^	3.5 (26.7)	9.1 (29.6)
Absolute change in body weight (kg) mean (±SD) ^e^	0.5 (3.6) ^b^	−0.1 (3.9)	1.0 (3.2)	0.6 (4.6) ^b,e^	0.1 (5.0)	1.1 (3.9)
Weight gain ≥ 1 kg *n* (%)	261 (39.0) ^b^	103 (33.9)	158 (43.3)	300 (42.0) ^b^	137 (36.1)	163 (48.7)
Increase in energy intake ≥ 250 kcal *n* (%)	186 (36.5) ^b^	72 (28.3)	114 (44.7)	229 (41.0) ^b^	100 (34.0)	129 (48.7)
Increase in protein intake ≥ 4 g/day	255 (50.1) ^b^	105 (41.3)	150 (58.8)	301 (54.1) ^b^	136 (46.4)	165 (62.7)
Increase in HGS ≥ 2 kg *n* (%) ^a^	226 (33.8)	99 (32.6)	127 (34.8)	154 (37.5)	76 (38.2)	78 (36.8)
Increase in HGS ≥ 4 kg *n* (%) ^a^	114 (17.0)	49 (16.1)	65 (17.8)	80 (19.5)	39 (19.6)	41 (19.3)

^a^ Data available for *n* = 200 control and *n* = 214 intervention. ^b^ Statistically significant difference between intervention and control. ^c^ HGS dataset: data available for *n* = 258 control and *n* = 259 intervention. Mortality dataset: data available for *n* = 318 control and *n* = 273 intervention. ^d^ HGS dataset: data available for *n* = 254 control and *n* = 259 intervention. Mortality dataset: data available for *n* = 294 control and *n* = 265 intervention. ^e^ Data available for *n* = 380 control and *n* = 335 intervention. Abbreviations: SD = standard deviation HGS = handgrip strength, kcal/day = kilocalories per day, g/day = gram per day.

**Table 3:** Overall treatment effect of nutritional intervention on ≥3 kg increase in handgrip strength (HGS) and mortality for older adults with (risk of) malnutrition, derived from GEE analyses; crude and adjusted for baseline variables.

	OR *	CI 95%
**Increase HGS ≥ 3 kg**		
Crude	1.146	0.822–1.598
Adjusted for age and sex at baseline	1.145	0.817–1.603
Adjusted for age, sex, BMI and HGS at baseline	1.110	0.766–1.588
**Mortality**		
Crude	0.790	0.450–1.387
Adjusted for age and sex at baseline	0.767	0.421–1.397
Adjusted for age, sex and BMI at baseline	0.780	0.416–1.461

* represents the treatment effect on the outcome measure derived from GEE. Abbreviations: GEE: generalized estimating equations, OR = odds ratio, CI = confidence interval HGS = handgrip strength, BMI = body mass index.

**Table 4:** Predefined subgroups analyses for baseline characteristics in older adults with (risk of) malnutrition.

	N Subjects C/I	OR *	CI 95%	*p* Interaction
**Increase HGS ≥ 3 kg**				
Age < 80 year	147/159	0.942	0.633–1.400	0.100
Age ≥ 80 year	157/206	1.348	0.930–1.954	
Women	188/234	0.694	0.694–1.618	0.925
Men	116/131	1.192	0.496–2.868	
HGS normal ^a^	109/122	0.844	0.392–1.818	0.655
HGS low	195/243	1.266	0.871–1.840	
BMI < 22	127/143	1.071	0.877–1.309	0.770
BMI ≥ 22	177/222	1.145	0.669–1.962	
Energy intake low ^b^	124/137	0.965	0.615–1.515	0.828
Energy intake high	136/127	1.834	1.002–3.356	
Protein < 0.8 g/kg/bw ^c^	75/83	0.641	0.373–1.102	0.119
Protein ≥ 0.8 g/kg/bw	185/181	1.907	1.234–2.947	
**Mortality**				
Age < 80 year	222/172	1.068	0.532–2.144	0.727
Age ≥ 80 year	180/188	0.653	0.318–1.342	
Women	259/232	0.631	0.341–1.165	0.072
Men	143/128	1.125	0.602–2.104	
HGS normal ^a^	66/74	1.741	0.383–7.920	0.474
HGS low	164/164	0.879	0.380–2.033	
BMI < 22	177/168	0.592	0.360–0.973	0.941
BMI ≥ 22	225/192	1.082	0.456–2.566	
Energy intake low ^d^	171/143	1.090	0.447–2.662	0.004
Energy intake high	161/155	0.756	0.340–1.678	
Protein < 0.8 g/kg/bw ^c^	142/116	1.725	0.641–4.639	0.052
Protein ≥ 0.8 g/kg/bw	189/180	0.536	0.211–1.362	

* represents the treatment effect on the outcome measure derived from GEE, adjusted for baseline ^a^ Cut-off values low HGS < 20 kg for women and <30 kg for men. ^b^ stratified at the median of 1682 kcal/day. ^c^ bodyweight was corrected to a BMI 22–27 kg/m^2^, ^d^ stratified at the median of 1682 kcal/day. Abbreviations: C/I control intervention, OR = odds ratio, CI = confidence interval HGS = handgrip strength, BMI = body mass index, g/kg/bw = gram per kilogram bodyweight.

## Data Availability

The deidentified participant data analysed for this project remain the property of the MaNuEL knowledge hub. Researchers should contact the original trial investigators directly for access to these data.

## Supplementary Materials

The following supporting information can be downloaded at: https://www.mdpi.com/article/10.3390/nu15092025/s1, Table S1: Characteristics of the included nutritional intervention RCTs.

## References

[1] Norman K., Pichard C., Lochs H., Pirlich M. (2008). Prognostic impact of disease-related malnutrition. Clin. Nutr..

[2] Milne A.C., Potter J., Avenell A. (2009). Protein and energy supplementation in elderly people at risk from malnutrition. Cochrane Database Syst. Rev..

[3] Cereda E., Pedrolli C., Klersy C., Bonardi C., Quarleri L., Cappello S., Turri A., Rondanelli M., Caccialanza R. (2016). Nutritional status in older persons according to healthcare setting: A systematic review and meta-analysis of prevalence data using MNA^®^. Clin. Nutr..

[4] Correa-Pérez A., Abraha I., Cherubini A., Collinson A., Dardevet D., de Groot L.C., de van der Schueren M.A., Hebestreit A., Hickson M., Jaramillo-Hidalgo J. (2019). Efficacy of non-pharmacological interventions to treat malnutrition in older persons: A systematic review and meta-analysis. The SENATOR project ONTOP series and MaNuEL knowledge hub project. Ageing Res. Rev..

[5] Borkent J.W., Naumann E., Vasse E., van der Heijden E., de van der Schueren M.A.E. (2019). Prevalence and determinants of undernutrition in a sample of dutch community-dwelling older adults: Results from two online screening tools. Int. J. Environ. Res. Public Health.

[6] Yang Y., Brown C.J., Burgio K.L., Kilgore M.L., Ritchie C.S., Roth D.L., West D.S., Locher J.L. (2011). Undernutrition at baseline and health services utilization and mortality over a 1-year period in older adults receiving Medicare home health services. J. Am. Med. Dir. Assoc..

[7] Kiesswetter E., Pohlhausen S., Uhlig K., Diekmann R., Lesser S., Uter W., Heseker H., Stehle P., Sieber C.C., Volkert D. (2014). Prognostic Differences of the Mini Nutritional Assessment Short Form and Long Form in Relation to 1-Year Functional Decline and Mortality in Community-Dwelling Older Adults Receiving Home Care. J. Am. Geriatr. Soc..

[8] Abizanda P., Sinclair A., Barcons N., Lizán L., Rodríguez-Mañas L. (2016). Costs of Malnutrition in Institutionalized and Community-Dwelling Older Adults: A Systematic Review. J. Am. Med. Dir. Assoc..

[9] Charlton K., Nichols C., Bowden S., Milosavljevic M., Lambert K., Barone L., Mason M., Batterham M. (2012). Poor nutritional status of older subacute patients predicts clinical outcomes and mortality at 18 months of follow-up. Eur. J. Clin. Nutr..

[10] Reinders I., Volkert D., de Groot L.C., Beck A.M., Feldblum I., Jobse I., Neelemaat F., de van der Schueren M.A., Shahar D.R., Smeets E.T. (2019). Effectiveness of nutritional interventions in older adults at risk of malnutrition across different health care settings: Pooled analyses of individual participant data from nine randomized controlled trials. Clin. Nutr..

[11] de van der Schueren M.A., Wijnhoven H.A., Kruizenga H.M., Visser M. (2016). A critical appraisal of nutritional intervention studies in malnourished, community dwelling older persons. Clin. Nutr..

[12] Munk T., Tolstrup U., Beck A.M., Holst M., Rasmussen H.H., Hovhannisyan K., Thomsen T. (2016). Individualised dietary counselling for nutritionally at-risk older patients following discharge from acute hospital to home: A systematic review and meta-analysis. J. Hum. Nutr. Diet..

[13] Baldwin C., de van der Schueren M.A., Kruizenga H.M., Weekes C.E. (2021). Dietary advice with or without oral nutritional supplements for disease-related malnutrition in adults. Cochrane Database Syst. Rev..

[14] Health Council of the Netherlands (2011). Undernutrition in the Elderly.

[15] Baldwin C., Smith R., Gibbs M., Weekes C.E., Emery P.W. (2021). Quality of the evidence supporting the role of oral nutritional supplements in the management of malnutrition: An overview of systematic reviews and meta-analyses. Adv. Nutr..

[16] Riley R.D., Lambert P.C., Abo-Zaid G. (2010). Meta-analysis of individual participant data: Rationale, conduct, and reporting. BMJ.

[17] Visser M., Volkert D., Corish C., Geisler C., Groot L.C., Cruz-Jentoft A.J., Lohrmann C., O’Connor E.M., Schindler K., Schueren M.A. (2017). Tackling the increasing problem of malnutrition in older persons: The Malnutrition in the Elderly (MaNuEL) Knowledge Hub. Nutr. Bull..

[18] Debray T.P.A., Moons K.G.M., Koffijberg H., Abo-Zaid G.M.A., Da Riley R. (2013). Individual Participant Data Meta-Analysis for a Binary Outcome: One-Stage or Two-Stage?. PLoS ONE.

[19] Debray T.P., Moons K.G., van Valkenhoef G., Efthimiou O., Hummel N., Groenwold R.H., Reitsma J.B. (2015). Get real in individual participant data (IPD) meta-analysis: A review of the methodology. Res. Synth. Methods.

[20] Bohannon R.W. (2019). Grip strength: An indispensable biomarker for older adults. Clin. Interv. Aging.

[21] Chan J., Lu Y., Yao M.M., Kosik R.O. (2022). Correlation between hand grip strength and regional muscle mass in older Asian adults: An observational study. BMC Geriatr..

[22] Cruz-Jentoft A.J., Bahat G., Bauer J., Boirie Y., Bruyère O., Cederholm T., Cooper C., Landi F., Rolland Y., Sayer A.A. (2019). Sarcopenia: Revised European consensus on definition and diagnosis. Age Ageing.

[23] Visser M., Mendonça N., Avgerinou C., Cederholm T., Cruz-Jentoft A.J., Goisser S., Kiesswetter E., Siebentritt H.M., Volkert D., Torbahn G. (2022). Towards developing a Core Outcome Set for malnutrition intervention studies in older adults: A scoping review to identify frequently used research outcomes. Eur. Geriatr. Med..

[24] Steiber N. (2016). Strong or weak handgrip? Normative reference values for the German population across the life course stratified by sex, age, and body height. PLoS ONE.

[25] Correa-Pérez A., Lozano-Montoya I., Volkert D., Visser M., Cruz-Jentoft A.J. (2018). Relevant outcomes for nutrition interventions to treat and prevent malnutrition in older people: A collaborative senator-ontop and manuel delphi study. Eur. Geriatr. Med..

[26] Kaegi-Braun N., Tribolet P., Baumgartner A., Fehr R., Baechli V., Geiser M., Deiss M., Gomes F., Kutz A., Hoess C. (2021). Value of handgrip strength to predict clinical outcomes and therapeutic response in malnourished medical inpatients: Secondary analysis of a randomized controlled trial. Am. J. Clin. Nutr..

[27] Berner L.A., Becker G., Wise M., Doi J. (2013). Characterization of dietary protein among older adults in the United States: Amount, animal sources, and meal patterns. J. Acad. Nutr. Diet..

[28] Beck A.M., Kjær S., Hansen B.S., Storm R.L., Thal-Jantzen K., Bitz C. (2013). Follow-up home visits with registered dietitians have a positive effect on the functional and nutritional status of geriatric medical patients after discharge: A randomized controlled trial. Clin. Rehabil..

[29] Beck A.M., Andersen U.T., Leedo E., Jensen L.L., Martins K., Rask K.O., Vedelspang A., Quvang M., Ronholt F. (2015). Does adding a dietician to the liaison team after discharge of geriatric patients improve nutritional outcome: A randomised controlled trial. Clin. Rehabil..

[30] de Jong N., Chin A., Paw M.J., de Groot L.C., Hiddink G.J., van Staveren W.A. (2000). Dietary supplements and physical exercise affecting bone and body composition in frail elderly persons. Am. J. Public Health.

[31] Manders M., de Groot C.P.G.M., Blauw Y.H., Dhonukshe-Rutten R.A.M., van Hoeckel-Prüst L., Bindels J.G., Siebelink E., van Staveren W.A. (2009). Effect of a nutrient-enriched drink on dietary intake and nutritional status in institutionalised elderly. Eur. J. Clin. Nutr..

[32] Neelemaat F., Bosmans J.E., Thijs A., Seidell J.C., van Bokhorst-de van der Schueren M.A. (2011). Post-discharge nutritional support in malnourished elderly individuals improves functional limitations. J. Am. Med. Dir. Assoc..

[33] Schilp J., Kruizenga H.M., Wijnhoven H.A., van Binsbergen J.J., Visser M. (2013). Effects of a dietetic treatment in older, undernourished, community-dwelling individuals in primary care: A randomized controlled trial. Eur. J. Nutr..

[34] Tieland M., van de Rest O., Dirks M.L., van der Zwaluw N., Mensink M., van Loon L.J., de Groot L.C. (2012). Protein supplementation improves physical performance in frail elderly people: A randomized, double-blind, placebo-controlled trial. J. Am. Med. Dir. Assoc..

[35] Stange I., Bartram M., Liao Y., Poeschl K., Kolpatzik S., Uter W., Sieber C.C., Stehle P., Volkert D. (2013). Effects of a Low-Volume, Nutrient- and Energy-Dense Oral Nutritional Supplement on Nutritional and Functional Status: A Randomized, Controlled Trial in Nursing Home Residents. J. Am. Med. Dir. Assoc..

[36] Cruz-Jentoft A.J., Baeyens J.P., Bauer J.M., Boirie Y., Cederholm T., Landi F., Martin F.C., Michel J.P., Rolland Y., Schneider S.M. (2010). Sarcopenia: European consensus on definition and diagnosis. Age Ageing.

[37] Lauretani F., Russo C.R., Bandinelli S., Bartali B. (2003). Age-associated changes in skeletal muscles and their effect on mobility: An operational diagnosis of sarcopenia. J. Appl. Physiol..

[38] Feldblum I., German L., Castel H., Harman-Boehm I., Shahar D.R. (2011). Individualized Nutritional Intervention During and After Hospitalization: The Nutrition Intervention Study Clinical Trial. J. Am. Geriatr. Soc..

[39] Cederholm T., Jensen G.L., Correia M., Gonzalez M.C., Fukushima R., Higashiguchi T., Baptista G., Barazzoni R., Blaauw R., Coats A. (2019). GLIM criteria for the diagnosis of malnutrition–a consensus report from the global clinical nutrition community. J. Cachexia Sarcopenia Muscle.

[40] Tsuboi M., Momosaki R., Vakili M., Abo M. (2018). Nutritional supplementation for activities of daily living and functional ability of older people in residential facilities: A systematic review. Geriatr. Gerontol. Int..

[41] Tieland M., Verdijk L.B., de Groot L.C., van Loon L.J. (2015). Handgrip strength does not represent an appropriate measure to evaluate changes in muscle strength during an exercise intervention program in frail older people. Int. J. Sport Nutr. Exerc. Metab..

[42] Norman K., Stobaus N., Gonzalez M.C., Schulzke J.D., Pirlich M. (2011). Hand grip strength: Outcome predictor and marker of nutritional status. Clin. Nutr..

[43] Verlaan S., Maier A.B., Bauer J.M., Bautmans I., Brandt K., Donini L.M., Maggio M., McMurdo M.E., Mets T., Seal C. (2017). Sufficient levels of 25-hydroxyvitamin D and protein intake required to increase muscle mass in sarcopenic older adults—The PROVIDE study. Clin. Nutr..

[44] Locher J.L., Vickers K.S., Buys D.R., Ellis A., Lawrence J.C., Newton L.E., Roth D.L., Ritchie C.S., Bales C.W. (2013). A randomized controlled trial of a theoretically-based behavioral nutrition intervention for community elders: Lessons learned from the behavioral nutrition intervention for community elders study. J. Acad. Nutr. Diet..

[45] Borkent J. (2022). Malnutrition during the Journey of Ageing.

