# Supplementary material for: Effects of Nutritional Interventions in Older Adults with Malnutrition or at Risk of Malnutrition on Muscle Strength and Mortality: Results of Pooled Analyses of Individual Participant Data from Nine RCTs
Source: Nutrients. 2023 Apr 22;15(9):2025. doi: 10.3390/nu15092025 (PMC10181186; doi:10.3390/nu15092025)
Supplement: Supplementary file 1 [file nutrients-15-02025-s001.zip › nutrients-2325042-supplementary.pdf]

Supplemental table S1. Characteristics of the included nutritional intervention RCTs

Study /Title	Age	Setting	N participants Control/ Intervention	Malnutrition inclusion criteria	Intervention	Duration	Reported intervention effect HGS	Reported intervention effect mortality	Reported compliance
Beck 2013 Follow-up home visits with registered dietitians have a positive effect on the functional and nutritional status of geriatric medical patients after discharge: a RCT	> 65y	Hospital	79/73	BMI <20.5 kg/m^2; and/or weight loss within the last three months; and/or reduced dietary intake in the last week; and/or serious ill (Level 1 screening in NRS2002)	Three individualized nutritional counselling visits by a dietitian complemented with three follow-up visits by general practitioners or three follow-up visits by general practitioners alone. ONS if needed.	12 weeks	HGS improved, N (%) 27 (45)/31 (50) P= 0.844	OR 0.60 (95% CI 0.17 to 2.13)	All three visits n (%)56 (97%).
Beck 2015 Does adding a dietician to the laison team after discharge of geriatric patients improve nutritional outcome: a RCT	> 70y	Hospital	37/34	WL >5% in 2 months; or BMI 18.5–20.5 kg/m^2 plus impaired general condition or food intake 25–60% of normal in past week or major disease (Level 2 screening in NRS2002)	Liaison-Team in cooperation with a dietician. The dietician performed 3 home visits, the first visit together with the discharge Liaison-Team, the remaining visits	12 weeks	Max kg, change median (95% CI) 0.5 / -0.4 P = 0.151	OR 0.323 (95% CI 0.060; 1.724).	Of the 31 participants who completed the study, 30 (97%) received the planned remaining two dietician visits.

					performed by a dietician alone. ONS if needed.				
Feldblum 2011 Individualized nutritional intervention during and after hospitalization: the nutrition intervention study clinical trial	> 65y	Hospital	181/78	MNA-sf score < 10; or WL ≥ 10% in previous 6 months.	Dietetic treatment in hospital; 3 home visits after discharge.	24 weeks	n.a.	3.8% had died in Group 1, versus 11.6% in Groups 2 and 3 (P=.046)	n.a.
de Jong 2000 Dietary supplements and physical exercise affecting bone and body composition in frail elderly persons.	> 70y	community-dwelling	40/59	BMI ≤25 kg/m^2; or recent WL.	2X2 design: 1) Enriched food 2) Exercise 3) Both 4) Neither. 2 products per day: 1 from a series of fruit products and 1 from a series of dairy products.	17 weeks	Not mentioned in the article although it was measured.	n.a.	Not mentioned in the article although it was measured.

Manders 2009 Effect of a nutrient-enriched drink on dietary intake and nutritional status in institutionalised elderly.	> 60y	Institutionalized	57/119	BMI ≤30 kg/m^2.	2 servings of 125ml of a complete nutrient-enriched dairy drink	24 weeks	Not mentioned in the article although it was measured.	n.a.	In the group of participants who completed the full protocol, median compliance was 78% (range 19–94%) and 80% (range: 44–92%) for intervention and placebo group, respectively.
Neelemaat 2011 Post-Discharge nutritional support in malnourished elderly individuals improved functional limitation.	> 60y	Hospital	105/105	BMI ≤ 20 kg/m^2; and/or ≥5% unintentional WL in the previous month and/or ≥10% unintentional WL in the previous 6 months.	2 servings of ONS, 6x Telephone counseling by dietitian. Energy and protein enriched diet (during the in-hospital period); Supplement of 400 IE vitamin D3 and 500 mg calcium per day.	3 months	change in kilo mean (±SD) 0.2 (5.6) / 1.0 (6.7). Difference (95% confidence interval)–0.8 (–3.0–1.5)	Not mentioned in the article although it was measured	Adherence to oral nutritional support was 80%,

Schilp 2013 Effects of a dietetic treatment in older, undernourished, community-dwelling individuals in primary care: a RCT.	> 65y	Community-dwelling	74/72	MUAC <25 cm; and/or ≥4 kg unintentional WL within the past 6 months.	Dietetic treatment, 1,000 mg calcium plus vitamin D 800 IU. ONS if needed.	3 months	(b = 0.49 kg, 95 % CI: -0.62–1.60)	Not mentioned in the article although it was measured.	n.a.
Stange 2013 Effect of a low-volume, nutrition- and energy-dense oral nutritional supplement on nutritional and functional status: a RCT in nursing home residents.	87 +/- 6 y	institutionalized	42/45	MNA score < 24 points; BMI ≤22 kg/m^2; a low food intake according to the nurses' perception; or WL≥ 5% in the past 3 months or ≥10% or more in the past 6 months.	2 servings of 125 ml ONS, Care personnel were instructed to encourage residents to consume the amount offered, and to support compliance.	12 weeks	HGS, kPa T1 32 (25-43) 34 (20-42) control 40 (35-54)43 (30-58) P = .407	Three participants of the IG (6.7%) and 7 of the CG (16.7%) died during the study period (P= .144).	Median compliance of 72.9% (23.6%-86.6%)
Tieland 2012 Protein supplementation improves physical performance in frail elderly people: a randomized, double-blind, placebo-controlled trial.	> 65y	community-dwelling	31/34	Fried criteria: prefrail when 1 or 2 criteria were applicable, frail when ≥3 criteria. (1) unintentional WL; (2) weakness; (3) self-reported exhaustion; (4) slow walking speed;(5) low physical activity.	2 servings, 250-mL containing 15 g protein.	24 weeks	After 24 weeks, handgrip strength in both groups had not improved (P > .05).	n.a.	After 24 weeks of dietary intervention, at least 92% of the provided drinks were consumed in both the protein and placebo supplemented groups.

Abbreviations: BMI = body mass index, MNA= mini nutritional assessment, MUAC = mid-upper arm circumference, ONS = oral nutritional support, WL= weight loss.
